# Shared Decision-Making During Initial Diagnostic and Treatment Planning Visits for Children with Autism Spectrum Disorder

**DOI:** 10.1097/DBP.0000000000000903

**Published:** 2021-01-13

**Authors:** Christina M. Mulé, Tara A. Lavelle, Samantha K. Sliwinski, John B. Wong

**Affiliations:** * Division of Developmental and Behavioral Pediatrics, Department of Pediatrics, Floating Hospital for Children at Tufts Medical Center, Tufts University School of Medicine, Boston, MA;; † Division of Developmental and Behavioral Pediatrics, Department of Pediatrics, Golisano Children's Hospital, University of Rochester, Rochester, NY;; ‡ Center for the Evaluation of Value and Risk in Health, Institute for Clinical Research and Health Policy Studies, Tufts Medical Center, Boston, MA;; § Division of Clinical Decision Making, Tufts Medical Center, Tufts University School of Medicine, Boston, MA.

**Keywords:** autism, shared decision-making, diagnosis, treatment planning

## Abstract

**Objective:**

Although shared decision-making (SDM) can improve patient engagement, adherence, and outcomes, evidence on the use of SDM within the context of autism spectrum disorder (ASD) initial diagnosis and treatment planning remains limited. The goal of this study was to objectively assess the occurrence of SDM in these visits and to compare this assessment with parent and provider perceptions of SDM in the same encounter.

**Methods:**

After audio-recording and transcribing initial clinical visits between parents (n = 22) and developmental behavioral pediatricians (n = 6) discussing the diagnosis of ASD and treatment options, we used the OPTION^5 Item^ scale to assess the occurrence of SDM. Afterward, parents and providers completed the OPTION^5 Item^, and parents also participated in a semistructured qualitative interview. Analysis consisted of descriptive statistics for OPTION^5 Item^ scores and a modified grounded theory framework for interviews.

**Results:**

Low levels of SDM were observed, with 41% of visits having no elements of SDM. On average, visits scored 1.1 of a possible 20 points on the OPTION^5 Item^ scale for SDM. By contrast, parents and providers indicated on the OPTION^5 Item^ scale that providers made a “moderate” to “skilled” effort to engage parents in SDM. Qualitative interviews with parents were consistent with their OPTION^5 Item^ ratings.

**Conclusion:**

The level of SDM determined by parent and provider reports was higher than the level of SDM determined by objective observation using a standard validated rating method. The findings reinforce the need for further research into barriers and facilitators of SDM methods and outcomes within ASD.

## Abstract 2

This article has supplementary material on the web site: www.jdbp.org.

As a growing public health concern that affects 1 in 54 children in the United States,^1^ autism spectrum disorder (ASD) is a lifelong condition with no cure, yet early access to and utilization of intervention services can greatly improve a child's development.^2–4^ The American Academy of Pediatrics (AAP) has emphasized the importance of early identification of ASD and early access to treatment to facilitate skill acquisition and improve overall outcomes.^5,6^ Treatment options include medications; behavioral interventions; speech, language, and occupational therapies; and complementary and integrative medicine.

After learning of a child's ASD diagnosis, parents often face challenging decisions when developing a treatment plan for their child^7^ that involve selecting, initiating, and coordinating therapeutic services across the medical, educational, and community sectors, often while still dealing with the difficult emotional experience of receiving a diagnosis of ASD for their child.^8^ Ideally, these treatment decisions should occur in a collaborative manner between families, whose expertise is their lived experience, constraints, and goals, and health care providers, whose expertise is in the care options and their evidence base.^9^

Shared decision-making (SDM) is a process by which patients, their families, and providers work together to choose a care plan that accounts for both the available evidence and the family's priorities and treatment preferences.^5,10,11^ The Institute of Medicine and the AAP advocate for SDM as a way to improve the quality of health care delivered in the United States.^12–14^ In over 100 randomized controlled trials in a variety of conditions,^15^ SDM has been found to help individuals improve their knowledge about the options, feel better informed with more clarity about their values, and probably be more active in the treatment decision-making process.^15^ In certain contexts, SDM may also increase patient adherence and improve outcomes.^15^ This is particularly important for families of children with ASD who already face substantial stressors related to their child's diagnosis, which can affect the quality of life and well-being of the whole family.^16,17^

Engaging families of children with ASD in SDM at their first diagnostic encounter, at which treatment options are initially discussed, could be a valuable opportunity to facilitate family-centered decision-making. However, to our knowledge, no objective data exist showing the extent or frequency of SDM in this setting. Previous studies relying on self-report from parents and providers have described limited SDM between parents of children with ASD and general pediatricians.^18^ In a subset of children seen for behavioral difficulties in the months and years after initial diagnosis, SDM occurred infrequently.^19^ Parents of these children reported leaving the visit with a sense of decisional conflict and uncertainty regarding the appropriateness of treatments recommended by their child's doctor.^19^

These previous studies show limited SDM in the care of children with ASD but do not provide information on discussions that occur at the time of initial disclosure of the diagnosis of ASD when parents and providers begin to plan treatments. The objective for this study was to objectively assess the frequency of SDM in the context of initial diagnostic and treatment planning visits for children with ASD and to compare this assessment with the subjective parent and provider perceptions of the utilization of SDM after that diagnostic encounter. This study seeks to determine whether these visits incorporate elements of SDM that may optimize family-centered care, parental engagement, and outcomes for children with ASD or whether future interventions are necessary to facilitate this practice.

## METHODS

### Setting

This study was conducted at a large academic medical center (AMC) in the Northeastern United States that contains a large multidisciplinary Division of Developmental and Behavioral Pediatrics (DBP) making over 200 new diagnoses of autism spectrum disorder (ASD) annually. All activities conducted as part of this research were approved by the AMC's Institutional Review Board with written consent from all participants before enrollment.

### Stakeholder Panel

At the outset of the study, we convened a 4-member stakeholder panel comprising 1 developmental behavioral pediatrician, 1 child psychologist/board-certified behavior analyst, 1 mother of a child diagnosed with ASD, and 1 father of a child with ASD. Throughout the study, the stakeholders met with the study team 4 times to provide feedback and guidance on the study design, including (1) recruitment, (2) adaptation of the measures, (3) design of the semistructured interview guide, and (4) interpretation of themes that emerged from semistructured qualitative interviews.

### Sample

#### Providers

Using purposive sampling, we recruited 8 providers meeting the following inclusion criteria: (1) specialty in DBP, (2) experience in diagnosing and treating children with ASD, (3) English speaking, (4) agreed to allow for the audio-recording of initial diagnostic and treatment planning visits with parents, and (5) agreed to assist the research team in the recruitment of 4 parents receiving a first-time diagnosis of ASD for their child. No incentives were given to providers for their participation in the study, and all 8 providers consented to participate. Two providers did not see any children who fit our inclusion criteria during the recruitment period, and therefore, our resulting sample only included 6 providers.

#### Parents

We approached and successfully recruited 24 parents based on the following inclusion criteria: (1) had a child between the age of 2 and 10 years who would be receiving a first-time diagnosis of ASD, (2) English speaking, (3) agreed to the audio-recording of initial diagnostic and treatment planning visit with provider, and (4) agreed to a follow-up interview. During the consent process, they were offered a $20 gift card for completing the study for their time and were informed that withdrawal from the study at any point would not affect the clinical care their child receives at the AMC.

### Procedures

All providers were blinded to the main objective of the research and were told that the research team was interested in better understanding the process of giving feedback to families after their child had undergone an autism evaluation. Providers notified the research team of every eligible family and when they would be in the DBP clinic for their initial diagnostic and treatment planning visit. In most cases, providers had assessed the child in an initial visit and requested a follow-up visit, and recruitment took place immediately before that follow-up visit occurred. One provider offered a “rapid screening” clinic where the assessment and feedback were offered in the same day. In this instance, the provider took a break to score assessment materials, solidify her clinical impressions, and notify the research team of the family's eligibility. During this break, the family was approached to participate in the study. Families were also blinded to the main objective of the study and were told the same information as providers regarding the purpose of the research. Once families completed the informed consent process, a member of the research team placed an audio-recorder in the treatment room to audio-record the visit and left.

Approximately 1 week after the visit, we conducted a 45- to 60-minute semistructured interview with one of the child's parents over the telephone in which we asked parents questions related to their experience receiving a first-time diagnosis of ASD for their child, how their provider engaged them developing a treatment plan for their child, and how their visit could have been improved on. We audio-recorded all interviews and transcribed the verbatim for analysis. Using a validated measure of shared decision-making (SDM), the Observing Patient Involvement OPTION^5 Item^ Scale (hereafter, “OPTION^5 Item^”),^20^ we also asked parents to verbally rate whether they believed that SDM occurred during their visit (see Figure, Supplemental Digital Content 1, http://links.lww.com/JDBP/A284, which shows the OPTION^5 Item^).

After providers completed recruitment of 4 families to the study, we asked them to complete the OPTION^5 Item^ to rate the extent to which they engaged in SDM with their patients' families, on average, over the duration of the study. To avoid any potential modifications in their behavior in response to being observed in their role in SDM (i.e., Hawthorne effect), providers were asked to rate their behavior only after all audio-recorded visits had been concluded.

### Measures

To assess the elements of SDM within the audio-recorded diagnostic and treatment planning visits, we used the OPTION^5 Item^, an objective measurement scale of SDM selected for its robust conceptual framework that includes the elicitation of patient preferences, an important omission in other measures of SDM. The measure is brief and rates providers on a scale from 0 (no effort) to 4 (exemplary effort) on 5 items of SDM, for a potential total score between 0 (worst) and 20 (best). The 5 items of SDM include (1) provider initiation of a discussion regarding the importance of the decision-making process in treatment selection, (2) provider reassurance that the patient and provider will work collaboratively as a team to make a decision, (3) provider's description of available treatment options and the pros and cons of each option, (4) provider engagement in a discussion of patient preferences and priorities, and (5) provider integration of patient preferences through collaboration and discussion (see Figure, Supplemental Digital Content 1, http://links.lww.com/JDBP/A284).^20^ This scale is more sensitive to the nuances of the decision-making context than alternative tools that use dichotomous ratings to describe the presence or absence of specific SDM elements.

The OPTION^5 Item^ was adapted for observing SDM for ASD treatment decisions, provider, and parent use, with the assistance of the stakeholder panel. The OPTION^5 Item^ has an accompanying manual with a detailed scoring guide and descriptors of each of the 5 elements of SDM, which assisted us in establishing acceptable inter-rater reliability.

For providers, the 5 items on the scale were reworded so that they could provide a self-report rating of how they engage families in SDM (e.g., for Item 1 of the OPTION^5 Item^, the new item read “Did you draw attention to…”). For parents, the items were reworded to reflect the performance of the provider (e.g., for Item 1 of the OPTION^5 Item^, the new item read “Did your provider draw attention to…”) and reduce the language demands (i.e., items were broken down and language was simplified where possible).

### Data Analysis

#### Quantitative

Two members of the research team (T.A.L. and S.K.S.) independently rated the audio-recording of each initial diagnostic and treatment planning visit for elements of SDM using the OPTION^5 Item^. Raters scored providers on a scale from 0 (no effort) to 4 (exemplary effort) for each of the 5 items of SDM, for a potential total visit score between 0 (worst) and 20 (best). Raters met to compare scores and discuss rating discrepancies until consensus was reached. We summarized the final OPTION^5 Item^ scores for observer (n = 22), parent (n = 22), and provider (n = 6) observations and computed means and standard deviations of scores for each of the 5 items within each group. We then compared parent, provider, and observer OPTION^5 Item^ scores.

In addition, raters independently evaluated each clinical visit for the presence or absence of 15 additional factors that promote parent engagement in the diagnostic and treatment decision-making process. These additional scoring factors captured parent-provider behaviors across 4 domains of family-centered care: (1) effective communication, (2) shared goal setting, (3) provider support, and (4) ASD- and treatment-specific parent education. Raters met to compare ratings and discuss rating discrepancies until consensus was reached. We summarized the mean and standard deviations for the scores of each of the 15 items across observations.

#### Qualitative

We analyzed parent interview transcripts using a modified grounded theory framework in which intensive group discussion between the research team and stakeholder panel regarding SDM in ASD care resulted in the identification of a priori interview themes, or codes (derived from literature review or stakeholder experience) and emergent themes, or codes (derived from the data).^21,22^ Codes, code definitions, and code examples were then summarized into a preliminary codebook. Using the preliminary codebook, 4 interviews were independently coded by a primary investigator (C.M.M.), and 2 separate research assistants who each coded 2 of those 4 interviews. The primary investigator (C.M.M.) then met with each research assistant to compare coded interviews, revise codes until consensus was reached, and solidify code definitions and interview example quotes for the final codebook.

Using the final codebook, all interviews were independently coded by 2 research team members. The primary investigator (C.M.M.) coded all 22 interviews, and each research assistant coded 11 interviews. To achieve coding agreement and fidelity, the research team met to review all coded interviews and discuss differences in coding until consensus was achieved.^22^ Once the team reached agreement on all of the coded interviews, transcripts were imported into Dedoose 8.0 (Los Angeles, CA), a web-based software program, for the analysis and summary of data.

## RESULTS

### Sample

Twenty-four parents participated in the audio-recorded visit portion of the study. Two parents did not complete a follow-up interview and were excluded from our analyses, leaving a final analytic sample of 22 parents (Table 1). Among the 22 parent participants, 5 were fathers and 17 were mothers. Most parents were born in the United States (64%), were native English speakers (73%), and used English as the predominant language spoken at home (68%). Fourteen parents completed high school or some college (64%), 4 completed college (18%), and 4 earned an advanced degree (18%). Of the children who were newly diagnosed, 18 (82%) were male and 4 were female. Based on parent report of child race/ethnicity, 54% were White, non-Hispanic; 9% were Black, non-Hispanic; 32% were Hispanic; and 5% were Asian. At the time of the diagnostic visit, the mean age of the children was 3.7 years (SD = 2.2 years), and the median age of the children was 3.0 years (interquartile range 2.0 to 4.8 years).

**Table 1.** Participant Demographics

Child demographics (n = 22)	
Age, yrs	
Mean	3.73
SD	2.23
Gender, n (%)	
Male	18 (82)
Female	4 (18)
Race/ethnicity, n (%)	
White, non-Hispanic	12 (54)
Black, non-Hispanic	2 (9)
Hispanic	7 (32)
Asian	1 (5)
Parent demographics (n = 22)	
Parent interviewed, n (%)	
Mother	17 (77)
Father	5 (23)
Country of origin, n (%)	
United States	14 (64)
Outside of the United States	8 (36)
Native language, n (%)	
Native English speaker	16 (73)
English as a second language	6 (27)
Language spoken at home, n (%)	
English	15 (68)
English and another language	5 (23)
Language other than English	2 (9)
Educational attainment, n (%)	
Less than high school	0 (0)
High school/some college	14 (64)
College	4 (18)
Advanced degree	4 (18)
Provider demographics (n = 6)	
Gender, n (%)	
Male	1 (17)
Female	5 (83)
Race/ethnicity, n (%)	
White, non-Hispanic	3 (50)
Black, non-Hispanic	0 (0)
Hispanic	0 (0)
Asian	3 (50)
Work experience, yrs, n (%)	
1–5	3 (50)
6–10	1 (17)
>10	2 (33)

Among the 6 developmental behavioral pediatricians who participated in the study, most were female (83%; Table 1). Half were non-Hispanic White, and the other half were Asian. Half of the providers were junior faculty at the academic medical center with 1 to 5 years of professional experience, 17% were mid-level faculty with 6 to 10 years of professional experience, and 33% were senior faculty members with over 10 years of professional experience.

### Quantitative Results

#### Observed Shared Decision-Making in Clinical Visits

Providers infrequently engaged families in the 5 key elements of shared decision-making (SDM) included in the OPTION^5 Item^ (Table 2). On a scale from 0 (no effort) to 4 (exemplary effort), clinicians were rarely observed to (1) discuss alternative treatment options (mean = 0.59, SD = 0.49), (2) convey their support for deliberation (mean = 0.05, SD = 0.21), (3) provide information about options (mean = 0.23, SD = 0.39), (4) elicit parent preferences (mean = 0.05, SD = 0.21), and (5) integrate parent preferences into decisions (mean = 0.18, SD = 0.35). The average total score for all 5 elements of SDM was 1.1 (SD = 1.1) across all 22 visits (possible range: 0–20), suggesting almost no SDM occurring in the entire sample of visits. Importantly, we found that in 41% of visits, providers provided no components of SDM during the office visit as rated with the OPTION^5 Item^.

**Table 2.** Observed, Parent-Reported, and Provider-Reported Shared Decision-Making Ratings

OPTION^5 Item^ Items^a^	Mean (SD)		
	Observed (n = 22)	Provider-Reported (n = 6)	Parent-Reported (n = 22)
1. For the health issue being discussed, the clinician draws attention to or confirms that alternate treatment or management options exist or that the need for a decision exists. If the patient rather than the clinician draws attention to the availability of options, the clinician responds by agreeing that the options need deliberation.	0.59 (0.49)	2.7 (0.39)	2.7 (1.1)
2. The clinician reassures the patient or reaffirms that the clinician will support the patient to become informed or deliberate about the options. If the patient states that they have sought or obtained information before the encounter, the clinician supports such a deliberation process.	0.05 (0.21)	2.8 (0.52)	3.2 (0.8)
3. The clinician gives information or checks understanding about the options that are considered reasonable (this can include taking no action) to support the patient in comparing alternatives. If the patient requests clarification, the clinician supports the process.	0.23 (0.39)	3.2 (0.41)	2.8 (1.0)
4. The clinician makes an effort to elicit the patient's preferences in response to the options that have been described. If the patient declares their preference(s), the clinician is supportive.	0.05 (0.21)	3.0 (0.32)	2.4 (1.2)
5. The clinician makes an effort to integrate the patient's elicited preferences as decisions are made. If the patient indicates how best to integrate their preferences as decisions are made, the clinician makes an effort to do so.	0.18 (0.35)	3.2 (0.26)	3.2 (1.0)
Total score (range: 0–20)	1.09 (1.11)	14.9 (1.42)	14.2 (3.7)

a OPTION^5 Item^ Items are scored 0 to 4, total scores can range from 0 to 20.

Although providers frequently engaged in many positive clinical behaviors during the clinical visit (e.g., 100% used plain/understandable language, 82% asked parents whether they had questions or concerns during the visit, and 68% provided reassurance to parents), they infrequently incorporated other elements of family-centered care, such as shared agenda setting (23%), parental goal elicitation (18%), and explanation of treatment evidence (14%; Table 3).

**Table 3.** Other Observed Family-Centered Care Behaviors

Other Observed Family-Centered Care Behaviors (n = 22)	n (%)
Effective communication	
Provider uses plain language during the visit	22 (100)
Provider uses the teach-back method	1 (5)
Provider asks parents whether they have questions during the visit	18 (82)
Provider recaps next steps at the end of the visit	14 (64)
Shared goal setting	
Provider uses shared agenda setting for the visit	5 (23)
Provider elicits parental goals for the child	4 (18)
Provider support	
Provider reassures parent(s)	15 (68)
Provider provides personal contact information	9 (41)
Parent education	
Provider asks parents to describe their understanding of autism	9 (41)
Provider explains the purpose and/or goals of treatment options	21 (95)
Provider explains the evidence supporting recommended treatment options	3 (14)
Provider provides follow-up resources and/or referrals	22 (100)

#### Parent and Provider Perceptions of Shared Decision-Making

Compared with the observed levels of low SDM, parents and providers perceived that SDM occurred more frequently in clinical visits (Table 2). On average, parents and providers indicated on the OPTION^5 Item^ that providers made a “moderate” to “skilled” effort to (1) discuss alternative treatment options (parent mean = 2.7, SD = 1.1; provider mean = 2.7, SD = 0.39), (2) convey their support for deliberation (parent mean = 3.2, SD = 0.8; provider mean = 2.8, SD = 0.52), (3) provide information about options (parent mean = 2.8, SD = 1.0; provider mean = 3.2, SD = 0.41), (4) elicit parent preferences (parent mean = 2.4, SD = 1.2; provider mean = 3.0, SD = 0.32), and (5) integrate parent preferences into decisions (parent mean = 3.2, SD = 1.0; provider mean = 3.2, SD = 0.26).

### Qualitative Results

Parents provided insight into their experience receiving a first-time diagnosis of autism spectrum disorder (ASD) for their child and how their provider engaged them in the diagnostic and treatment planning process. From the semistructured interviews, we identified 7 core themes elaborated further below: (1) pragmatic expectations for the diagnostic feedback visit, (2) clear and thorough explanation of the diagnosis, (3) mixed reactions to the diagnosis, (4) mixed dialogue with providers after diagnosis, (5) varied methods for treatment plan development, (6) lay explanation of treatment plan, and (7) understanding of treatment plan. Finally, we share parent recommendations for ways to improve the visit.

#### Pragmatic Expectations for the Diagnostic Feedback Visit

Most parents reported that their expectations for the diagnostic feedback were met (79%), and approximately half described having had previsit pragmatic expectations (45%). Namely, they described being interested in gaining diagnostic clarification and a better understanding of how to help their child make developmental progress (see Table 4 for a sample of illustrative quotes). Parent responses did convey “process expectations” for how families would engage with their providers and how they would arrive to a satisfactory treatment plan for their child.

**Table 4.** Themes Identified from Parent Interviews and Illustrative Quotes

Themes	Parents' Comments
Receiving the diagnosis	
Pragmatic expectations for the diagnostic feedback visit	My only expectations were really, what is his diagnosis, how can I help him, and what should I expect. My expectation was just, I don't really know, to be honest with you, I think I just wanted to have a more clear-cut answer as to what was happening in so that, the proper course of treatment, the proper course of intervention could be applied, and as soon as possible. All I want is for him to say like this is the problem and this is how we're going to fix it.
Clear and thorough explanation of the diagnosis	
Layperson's terms	She just, she told us kinda about how she diagnoses it, and that it’s more based on—she kinda explained it like Swiss cheese pretty much, and the holes were the deficiencies in someone, and how autism is diagnosed based on those holes, and that [my son] had these great, great things about him, but the holes, his deficiencies, were so large that he fit for that, I'm paraphrasing, she said it in a great way, but she basically said he qualified, or he fit the diagnosis because of his, the things that he—his holes. She explained everything to my understanding, and if I didn't understand what she was saying, or had questions about it, she would either re-explain it in a way I understood, or she would answer the question. …everything she said she said in a way we could understood.
Detailed	I think her feedback visit was perfect the way she talk with us. She went with us point by point and show us why she had the diagnosis. It was really, really good. The way she talk to us and she show us why, why she has this problem. She was really comprehensive. I mean she went in depth of this is why I think this and that's why I did that test and that test and you know, he stared this way, he stared that way, I also noticed this, things that I didn't even notice.
Reaction to diagnosis	
Prepared	I wasn't surprised by what she told me; I've known for a while, waiting for the appointment. Well obviously, I'm sad and I'm devasted for him, but it's not surprising, I had time to kind of prepare. I kind of suspected that, and it didn't come as a surprise, it didn't—I wasn't surprised, it didn't take me by storm, but I knew it could be something—it could be a diagnosis that could be given, so it wasn't a surprise for me.
Shocked	I wasn't prepared for this at all, now I have to do more work than what I'm doing right now. I really didn't think he was, so I was shocked. Yeah, I was shocked. I was just—I really didn't think it was going to be that. But, I think, I don't know—it might be hard too since it's your son, so there's always been a special connection between me and him, and I have always doted on him, and maybe it’s that, that parental blindness.
Mixed dialogue with providers after diagnosis	
None	I honestly didn't have any questions because it was something that was long coming, and his explanation was something that I needed to hear and I needed someone to say anything at all to kind of fill in the kind of questions or spots in my head that I was kind of filling in myself. No, I didn't have any questions because I really wanted to, for him to, he's the doctor in this case to say, this is the issue so he has this, this and that—like be specific so that I know so I can take him to where the situation can be fixed. I didn't really have many questions; she more—all the questions that I would've had she answered just telling me about it.
Treatment	I just mainly questioned what the next steps would be, as it comes to medicine, or therapy, or what my next course of action would be. I think I more just wanted to get her take as to specifically, not what she was recommending in terms of the treatment options, but more about what she was hoping those treatment options would provide for him. So I know what ABA is, but what specifically was she hoping they'd look at and worked with him on right away. I did question why he would need ABA for what targeted behavior because my experience with ABA, because it is such an intensive treatment, it's for you know intense behaviors, severe behavior.
Varied methods for treatment plan development	
Prescriptive	No, I mean… what I know is that, number 1, we, we have a kid who needs help, and the doctors have diagnosed—I mean we have a kid who we don't know why he doesn't speak, or do things like the other normal kids do—so the doctors, we presented our problems to the doctor, or our concerns to the doctor, and they doctors diagnose, use their professional skills and abilities and knowledge to diagnose the issue—the problem. So, to me, it was my job, or our job as parents, to listen from the professional, and then we were very much involved when they were asking questions. I just feel like I didn't do my part to work with her, so it wasn't really her responsibility—her fault at all, it was just that I didn't really assert myself and say that I don't think that'd work, you know? I definitely let him take the reins because he knows far more about this than I do, so I'll take any advice that he has to give me.She did write down things and she did write out a plan on a piece of paper, but it was kind of hard to understand what she was wanting me to do, because she kept on mentioning ABA and I don't know what the hell an ABA is. So she communicated [the treatment plan] and we agreed with what she decided.
Collaborative	She told me what she thought her—what the next steps should be, but she also took my input into play and kind of combine the 2. We work together, spending time talking about how we want [my daughter] to have more of an in-school for like occupational therapist and speech therapist and wants her to go like summer school and everything. We worked together, yeah because when she said this and this and this that's my recommendation, I said like yeah, he's in early intervention right now, but we can talk to them for increase that, that's the way they, this is like she recommended, and they're going to work with the ABA for us, so she's like okay, that's going to be a great idea, so we talking about the treatment, so that's good, because she include us in that.
Lay explanation of treatment plan	She did say that if you think early intervention helped, you'll see a lot, a lot of progress of the 20 wk of more intense therapy. She said that that is going to be very helpful—that all I do is that they help him doing a task and they divide it by, like mini tasks, meaning that to do something, they teach him how to do it in baby steps. I do remember her saying that, that, because he was so young, and if we got all these treatment options on board, that most likely he would have, you know, he would make huge improvements. She told me they're—I think I remember she did tell me they are the best, and they are professionals, let me see [rustling of paper] actually I have a piece of paper that tells me more about ABA. She didn't really go into research, like this research has said that ABA has been effective for kids on the spectrum. Not that I could remember.
Understanding of the treatment plan	
Strong understanding	I've already heard about it and researched it a little bit myself. I feel like I was already familiar with it. I already picked an ABA company I wanted to use, so I don't think she needed to go into too many details because I kind of already knew. Yeah, we'd already done the reading by the time we had the second meeting, we had both already read the entire packet we were given, which was a 34-page packet, something like that. So, when she mentioned ABA, she asked if we knew about what ABA is and I said yes, and my wife knows it as well, so she didn't have to go into what ABA was.
Limited understanding	She asks us to look in the website about the Autism Speaks, and she gave us folder with some explanation, and—but it was not so much more than that. I still don't know what ABA is. I try to learn it after I arrive at home, but I think it's so much information to do, to give us in just 1 hr. She doesn't have time to explain what the therapy does, and I think she did a really good job. I think now it's my job to try to look into what she said, and she can't explain everything in 1 hr. It was kind of hard to understand what she was wanting me to do, because she kept on mentioning ABA and I don't know what the hell an ABA is. It takes me a while to learn about things like this. I don't really get it. It's all still confusing to me. It's probably just me though.
Recommendations for improved visits	It would be cool if they gave you something that could give you a clearer explanation about what's happening with the biology and genes of autism, what's going to be different in your family, but something that's done in a clearer way, kind of like an engaging way. Yeah, something more approachable, yes. I'm obviously being a bit facetious when I go all the way to the cartoon drawing, you know. But yeah, because the packet's informative, don't get me wrong, it's, it's very dense… I think it was so much information in just, I think it was 1 hr, I'm not sure. But it could be, if it was one, I'm not sure if it was 1 hr, but if it was 1 hr it could be one and a half hours. I think it would be a good idea [to have a second visit]. Yeah, because for me, it was I didn't have time for something what to do until now, because I was—it was shocking. But for, I think for parents that are expecting to have their child [diagnosed], maybe 1 hr would be enough because they already know what the child has and could use the time just for the treatment. Yeah, when she was telling me everything, it was a little bit hard… I understood what she was saying, but it was a lot of information. It's overwhelming. So, I'm kind of in this spot where I'm not really sure what to do and I wish I had someone to help me and guide me through this, because I have no clue.

#### Clear and Thorough Explanation of the Diagnosis

Most parents (95%) spoke favorably of their providers' approach to giving a diagnosis of ASD. Specifically, many parents reported that their provider used language that was easily interpretable for them or that their provider used strategies to help improve their comprehension (e.g., used analogies, clarified through repetition, invited parents to ask questions, etc.). In addition, parents noted that their providers were detailed in their approach to giving the diagnosis and frequently linked diagnostic criteria to behavioral observations during standardized testing and highlighted subtle behavioral observations (e.g., complex finger mannerisms or visual inspection) that are frequently overlooked by untrained individuals.

#### Mixed Reactions to the Diagnosis

Parents described having a range of emotions when receiving the initial diagnosis of ASD for their child. However, parents ranged from feeling either prepared for the diagnosis (59%) or shocked by the diagnosis (36%). Those who described feeling prepared frequently cited having been told by other professionals (e.g., early intervention or pediatrician) that their child would likely meet criteria for ASD and having had time to prepare themselves to receive the diagnosis (e.g., do research and have discussions internally with the family).

By contrast, some parents described feeling shocked by the diagnosis, referencing (1) an inability to see potential signs of ASD objectively because they are the parents of their child, (2) misattribution of symptoms to other neurodevelopmental disorders (e.g., attention-deficit/hyperactivity disorder), and (3) overwhelm related to the prospect of treatment. These parents frequently described becoming emotional during their diagnostic feedback visit or immediately thereafter (e.g., crying in the car), with some also becoming emotional during the semistructured interview occurring approximately 1 week after their initial diagnostic and treatment planning visit.

We performed a sensitivity analysis to examine whether the occurrence of SDM varied between visits in which the parents were “prepared” for the diagnosis compared with those who were “shocked” by the diagnosis. We found no significant differences in OPTION^5 Item^ scores between the 2 groups (mean = 1.1 for the “prepared” group and 1.0 for the “shocked” group).

#### Mixed Dialogue with Providers During the Visit

Parents reported mixed engagement with their provider after the diagnosis was shared during the visit. Just over half of the parent participants reported that their provider offered sufficient details in the diagnostic feedback visit and required little additional dialogue (55%). Parents who were more prepared for the diagnosis reported that their provider merely filled in the gaps in their own understanding of their child, whereas parents who felt less prepared or even shocked indicated that the emotional experience of learning of the diagnosis interfered with their ability to generate questions during the visit. In addition, these same parents frequently reported not knowing enough about ASD to formulate relevant questions. Instead, they felt it better to actively listen and absorb as much as they could from their provider. Finally, when parents reported asking additional follow-up questions (45%), their questions and dialogue centered on the course of treatment for ASD (e.g., method and goals of treatment; 89%) and ASD trajectory over time (32%) and less frequently on the causes or cause of ASD (18%).

#### Varied Methods for Treatment Plan Development

Treatment plans consisted of standard treatment approaches to ASD (e.g., applied behavioral analysis [ABA], speech and language therapy, occupational therapy, etc.) in which services were delivered via individualized family service plans, individualized education plans, or at private outpatient therapy centers. As described by the parents, treatment plan development consisted of 2 primary approaches: collaborative (68%) or prescriptive (32%).

Most families reported feeling as though the process of developing a treatment plan was collaborative. They frequently related that their provider would share their treatment recommendations, check in with the family, and incorporate their ideas/feedback into the plan (e.g., concerns about the frequency/duration of treatment).

By contrast, parents who described the process as being more prescriptive frequently reported that they preferred that their provider “take the reins” because they felt as though the provider had the expertise to guide the treatment. These parents felt most comfortable answering provider questions and listening. In addition, some parents reported feeling uncomfortable asserting their opinions when they disagreed with their provider, so they presented themselves as being agreeable and accepted responsibility for the limited provider-family collaboration. Parents also reported that their provider seldom asked them about short- and long-term goals for their child and thus were not able to link recommended interventions to their goals.

#### Lay Explanation of the Treatment Plan

Parents were asked about their provider's approach to describing the available treatment options for ASD. Parent narratives frequently depicted provider explanations as comprehensible (e.g., intensive treatment will lead to greater gains; the earlier the intervention, the better; etc.) but lacking in evidence base. Specifically, only a small portion of parents (27%) could recall their provider sharing with them the evidence base for the recommendations they shared.

#### Understanding of the Treatment Plan

Just over half of the parent participants reported that they had a strong understanding of the treatment plan discussed with their provider at the end of their initial diagnostic and treatment planning visit (59%). Parents credited their understanding to their provider's ability to explain the treatment plan in lay terms, but many of these parents referenced having had previous knowledge and experience from other professionals who had assisted in their child's treatment. In addition, some parents reported that they were able to enhance their knowledge by reading about treatment options for ASD before their visit.

A smaller proportion of parents discussed being confused about their child's treatment plan, such as about what ABA is and what it targets. Parents frequently accepted responsibility for their lack of understanding, stating that they might be slower to absorb information. Strikingly, parents excused providers for having overlooked their information needs, stating that they have too much to cover in a 1-hour visit and that they will have to do additional reading to better educate themselves. In addition, some parents also referenced being connected to other support staff (e.g., social workers and resource specialists) who could help them improve their knowledge.

#### Recommendations

By and large, families were satisfied with their experience receiving a first-time diagnosis of ASD, and most parents spoke favorably of their providers, stating that there was nothing that could be done to improve their visit. Even so, many parents reported that there was a lot of information presented to them in a short period of time and that it was challenging to absorb everything shared with them. Some parents commented that the process could have been strengthened with (1) additional time (e.g., adding 30 minutes to the visit); (2) additional follow-up visits that would allow families to process the information, do some research, and come back with additional follow up questions; and (3) additional tools or resources that could make the information shared in the initial diagnostic and treatment planning visit more understandable.

## DISCUSSION

This is the first study to objectively assess shared decision-making (SDM) during the initial diagnostic and treatment planning visits for children with autism spectrum disorder (ASD) and elicit parent and provider perceptions of SDM during these visits. We observed low levels of objective SDM process measures during these visits, with 41% of visits having no SDM within the entire encounter. On average, visits scored 1.1 of a possible 20 points on the OPTION^5 Item^ for SDM. By contrast, however, parents and providers indicated on the OPTION^5 Item^ that providers made a “moderate” to “skilled” effort to engage parents in SDM, with mean reported total scores of 14 by parents and 15 by providers (of 20). Qualitative interviews with parents conveyed information that was consistent with these parent and provider OPTION^5 Item^ ratings, with most parents reporting that they felt their provider took a collaborative approach to treatment planning by checking in with the family throughout the visit and incorporating parent perspectives and feedback into the plan.

This inconsistency between parent and provider perceived levels of SDM and observed levels of SDM may be explained in part by the other important elements of family-centered care that Division of Developmental and Behavioral Pediatrics (DBP) provided throughout the visit. For example, we observed that providers communicated effectively with the parents, consistently using plain language to describe the diagnosis and treatment, and often attempted to engage parents in the conversation by asking them whether they had questions or concerns throughout the visit. Qualitative findings support these observations, with almost all parents conveying that they liked their providers approach to giving them diagnostic and treatment information for their child and appreciated their attempts to provide clear and comprehensive explanations throughout the visit.

Our results are consistent with previous studies that show infrequent SDM process measures among providers and parents of children with ASD,^18^ even when compared with children with other developmental disabilities.^23,24^ Using data from the 2009–2010 National Survey of Children with Special Health Care Needs, Lipstein et al.^24^ found that parents of children with ASD were less likely than parents of children with asthma or attention-deficit/hyperactivity disorder to report that their child's doctors had engaged them in SDM over the past 12 months. Using this same data source, Hubner et al.^23^ found that SDM also occurred less frequently with children with ASD versus children with cerebral palsy or Down syndrome. Moreover, SDM occurred less frequently in visits with children with greater functional impairment. These studies draw on the strength of a national survey but use survey questions that ask about SDM in all health care visits over the past year, rely on parent self-report (which is not free of recall bias), and do not explicitly ask parents about their experiences with SDM in the context of making ASD-related treatment decisions. One additional study used the OPTION^5 Item^ to observe how often SDM occurred between parents of children previously diagnosed with ASD and DBP in visits to discuss treatment options for challenging behaviors.^19^ As in our study, SDM occurred infrequently in this context as well.

Unlike these previous studies, our study combines the power of observed data with contextual information from parent and provider surveys and parent interviews. Our conversations with parents indicate that although they felt that their needs were met during the visit, they approached the visit simply expecting to understand what the diagnosis was and how they could help their child improve. Parents generally had few expectations on how they would engage with their clinician to develop a treatment plan. Although most parents felt that the treatment planning was collaborative, some parents felt uncomfortable being an active player in their child's treatment planning.

Another important barrier to SDM in this context is visit time—providers are allotted 1 hour to both provide parents with their child's diagnosis and start treatment planning. This time may not be sufficient to fully accomplish everything the provider would like to do during that visit. In addition, the inconsistency between provider ratings and observed ratings of SDM indicate that providers may not be fully aware of all of the components involved in SDM.

This highlights opportunities to facilitate SDM during these visits and suggests using tools and resources that could guide parents and providers through important aspects of SDM in the context of the diagnostic and initial treatment planning visit. SDM tools such as decision aids (DAs) have been incorporated into clinical decisions for surgery, screening, genetic testing, and medication treatments,^15^ but none exist for use in diagnostic and treatment planning visits for ASD. A Cochrane review of 105 randomized studies of DAs found that DAs improve decision quality, reduce the proportion of individuals undecided about treatment plans, and impact treatment choices.^15^ A small but important group of parents in our study indicated that they did not understand their child's treatment plan following the visit, exposing an important gap in the current standard of care. The use of a DA in ASD treatment planning visits has the potential to provide parents with the knowledge they need identify and implement the treatment choices that are best suited for their child and family.

### Limitations

Several limitations for this study exist. First, although our sample of providers was purposive, providers all worked within the same academic medical center and their approach to ASD diagnosis and treatment planning may not be representative of providers in our or other regions of the United States or other practices in academic or nonacademic settings (e.g., community health centers or private practice). In addition, our sample of providers consisted of DBP only because they diagnose ASD most frequently; however, further research could explore whether and how other commonly consulted professionals (i.e., psychologists and neurologists) engage their patient's families in SDM as well.

Second, the sample of parent participants was small and limited to English-speaking families only, limiting the generalizability of our results. Multisite studies that can increase the size and diversity of the current sample would enhance generalizability and strengthen conclusions using inferential statistics and recommendations for future practice. In addition, 2 parents consented to the study and had their child's visit audio-recorded but did not complete their follow-up interview and were excluded from the study. We did not score these 2 visits using the OPTION^5 Item^, and we do not know whether there were characteristics of these 2 families or their child's visit that are different from those who did complete the follow-up.

Third, although the research team was not involved in the clinical care of the families that participated in this study, it is possible that parents felt the pressure to rate their providers and clinical experience more favorably on OPTION^5 Item^ because of perceived affiliation. In addition, because provider and parent ratings on the OPTION^5 Item^ were not collected immediately after the clinical encounter and relied on their memory of the visit, it is possible that participants did not accurately recall the events of the clinical encounters and therefore inaccurately provided OPTION^5 Item^ ratings. In addition, we asked providers to rate the extent to which they engaged in SDM with parents on average over the duration of the study, which may have exacerbated recall bias and caused us to miss important variation across visits. Future study designs should consider more autonomous and immediate ways of collecting these data to mitigate the potential impact of response and recall bias.

Finally, although the current study gathered provider perceptions of their ability to engage families in SDM via the OPTION^5 Item^, it did not collect qualitative perceptions from providers that would offer additional insights (e.g., how do providers see their role in SDM? What are the challenges from their perspective in engaging families in SDM?). Qualitative data that capture provider perspectives will be particularly useful as the field considers interventions that will enhance bidirectional conversations between providers and families.

## CONCLUSIONS

During initial diagnostic and treatment planning visits for children with autism spectrum disorder, the level of shared decision-making (SDM) determined by parent and provider reports was higher than the level of SDM determined by objective observation using a standard validated rating method. Interventions that may improve SDM in these settings include decision aids (DAs), which can facilitate SDM and empower providers and parents to work together to develop a treatment plan that is best suited for the child and family's needs. Future work is needed to develop a DA and determine whether this tool can improve providers' ability to engage parents, increase parental knowledge, reduce decision conflict about treatment, and clarify treatment preferences.
